# Predictors for the restoration of the sagittal spinal malalignment in patients with lumbar stenosis after short-segment decompression and fusion surgery

**DOI:** 10.1186/s12891-022-05666-2

**Published:** 2022-07-26

**Authors:** Rufeng Huang, Fumin Pan, Weiguo Zhu, Chao Kong, Shibao Lu

**Affiliations:** 1 Department of Orthopedics, Xuanwu Hospital, Capital Medical University, Changchun Rd. 45, Beijing, 100053 P.R China; 2 National Clinical Research Center for Geriatric Diseases, Beijing, China

**Keywords:** Lumbar spinal stenosis, Sagittal malalignment, Age, Sex, Short-segment, Decompression and fusion surgery

## Abstract

**Background:**

To explore the predictors for the restoration of the sagittal spinal malalignment in the elderly patients with lumbar spinal stenosis (LSS) after short-segment decompression and fusion surgery.

**Methods:**

We retrospectively reviewed 82 LSS patients with sagittal malalignment (SVA ≥ 50 mm or PT ≥ 20° or PI-LL ≥ 20°) who underwent short-segment decompression and fusion surgery between January 2019 and March 2021. Patients’ characteristic, radiographic and paravertebral muscle parameters were assessed. The patients were divided into group A (postoperative malalignment) and B (postoperative alignment) according to whether the postoperative restoration of the sagittal alignment was achieved.

**Results:**

There existed more males in group B than in group A (*p* = 0.002). The age of group A (73.36 ± 8.02) was greater than that of group B (69.08 ± 6.07, *p* = 0.009). Preoperative PT in group A (27.40 ± 5.82) was greater than that in group B (19.30 ± 7.32, *p* < 0.001). The functional cross-sectional area (fCSA) in group A (28.73 ± 4.23) was lower than that in group B (36.94 ± 7.81, *p* < 0.001). And the fatty infiltration rate (FI) of group A (27.16% ± 5.58%) was higher than that of group B (22.61% ± 5.81%, *p* = 0.001). The fCSA was negatively correlated with the postoperative PT and PTr (*p* < 0.05).

**Conclusion:**

Stronger lumbar paravertebral muscles, smaller preoperative PI, PT or PI-LL, male and younger age are the predictors for the restoration of the sagittal spinal malalignment in the elderly LSS patients after short-segment decompression and fusion surgery.

## Background

Lumbar spinal stenosis (LSS) is one of the most common degenerative spinal diseases in the elderly population [1, 2]. With the aging process, the degeneration of the intervertebral disc, the thickening of the ligamentum flavum and facet joints would eventually lead to the narrowing of the spinal canal [3]. These pathological changes could cause clinical symptoms of low back or leg pain, or neurogenic claudication, which would severely impair the quality of life [4]. If the symptoms don’t relieve after conservative therapy, surgical management should be performed and the decompression and fusion surgery is generally considered the golden standard [5, 6].

Despite LSS, adult spinal deformity is also one of the most important issues impacting the quality of life [7]. Among all deformities, the sagittal spinal malalignment has been shown to be significantly correlated with a worse health status, including the physical and mental health in adults [8]. For correcting the spinal malalignment, long-segment fixation and pedicle subtraction osteotomy is one of the most common surgical methods [9], which might however cause many problems including the long operation time, large amount of blood loss, high incidence of postoperative complications, and great cost of instrumentation and reoperation [10, 11].

Unfortunately, some patients suffer not only from LSS, but coexist with sagittal spinal malalignment. After decompression at the stenosis levels, it is difficult to decide whether to correct the sagittal malalignment with long-segment osteotomy and fusion surgery, as some patients were reported to restore their normal sagittal morphology only through short-segment decompression and fusion surgery and others were not [12, 13]. The trunk muscle condition and the severity of preoperative sagittal malalignment would be the potential predictors. Nevertheless, few studies have figure out in this field, especially the influence of the lumbar paravertebral muscles on the postoperative sagittal spinal balance.

The purpose of this study was to explore the preoperative predictors for the restoration of the sagittal malalignment in patients with LSS after short-segment decompression and fusion surgery. Furthermore, we aimed to provide some preoperative evaluation parameters for spine surgeons in the selection of surgical methods to correct sagittal malalignment.

## Materials and methods

### Patients

The hospital ethical review board approved this retrospective study. The medical records of patients who underwent short-segment (≤ 3 levels) posterior lumbar interbody fusion (PLIF) or transforaminal lumbar interbody fusion (TLIF) with pedicle screw fixation due to LSS with sagittal malalignment between January 2019 and March 2021 were retrospectively reviewed. The inclusion criteria were as follows: (1) age between 60 and 90 years; (2) the symptoms were mainly from LSS and invalid conservative treatments; (3) a diagnosis of LSS with sagittal malalignment (SVA ≥ 5 cm or PT ≥ 20°, or PI –LL ≥ 20°) [14]; (4) patients who were followed up for more than 3 months postoperatively. The exclusion criteria were: (1) inappropriate radiography; (2) lumbar spondylolisthesis (slippage of 1 vertebra over subjacent vertebra); (3) any serious medical comorbidity such as sepsis or malignancy; (4) previous lumbar instrumented surgery and (5) with neuromuscular diseases.

### Radiographic assessment

**Fig. 1:**
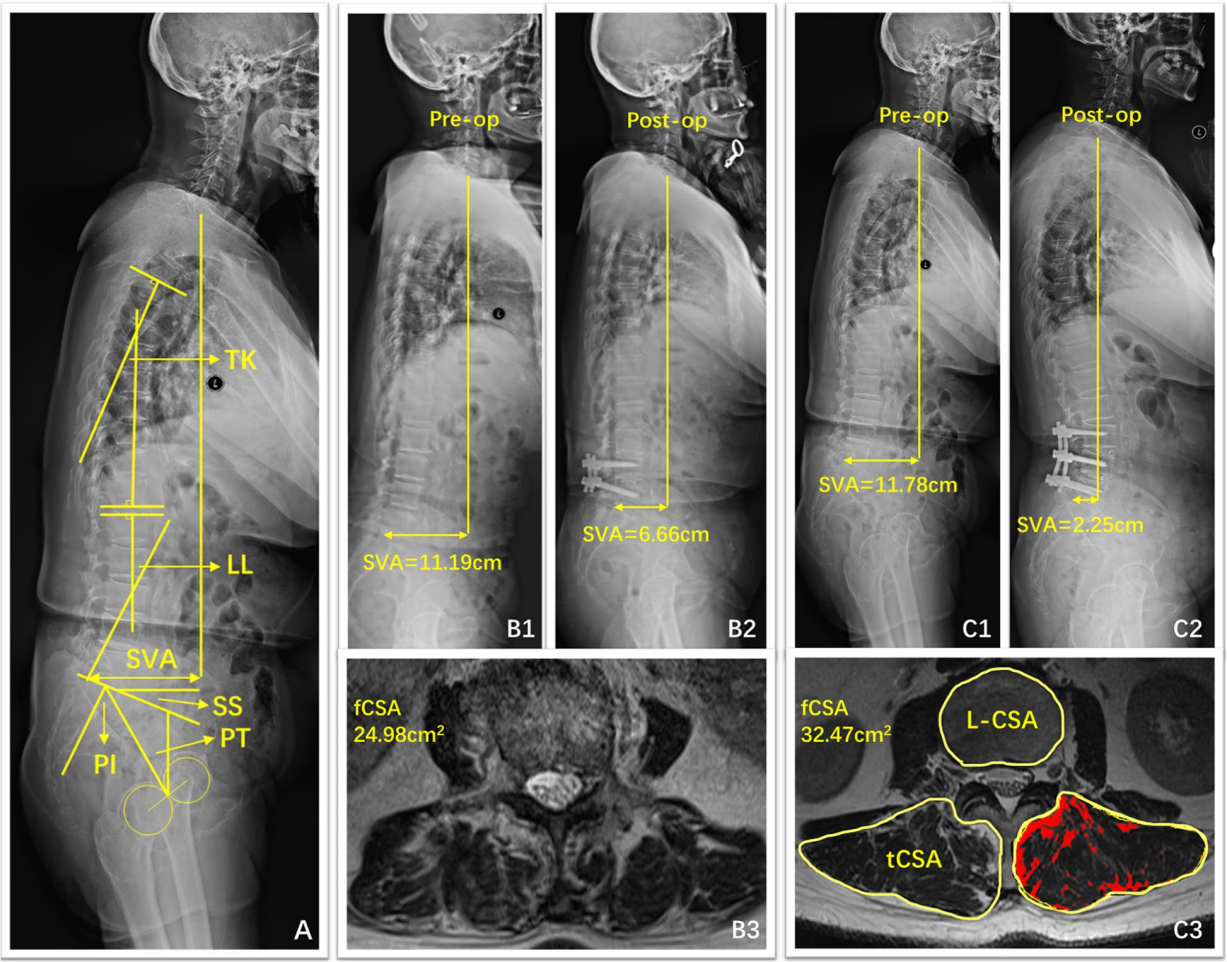
(**A**) The depicting of the sagittal spinopelvic parameters. TK: thoracic kyphosis; LL: lumbar lordosis; SVA: sagittal vertical axis; SS: sacral slope; PT: pelvic tilt; PI: pelvic incidence. (**B**) A representative case for group A of a 80 years old female patient, with B1 for the preoperative lateral radiograph, and B2 for the 1-year postoperative lateral radiograph and B3 for the preoperative muscle assessment. (**C**) A representative case for group B of a 70 years old female patient, with C1 for the preoperative lateral radiograph, and C2 for the 5-month postoperative lateral radiograph and C3 for the preoperative muscle assessment

Pre- and postoperative full-spine radiographs were performed using 36-inch-long digital lateral radiographic films with patients looking forward while trying to maintain a horizontal gaze and with their arms flexed, hands placed on their clavicles, and knees extended. Sagittal spinopelvic parameters, including sagittal vertical axis (SVA), lumbar lordosis (LL), pelvic tilt (PT), sacral slope (SS), and pelvic incidence (PI) were measured on full-spine lateral x-rays (Fig. 1A). PI minus LL, PT/PI (PTr) were also calculated [15, 16]. The patients were divided into two groups according to the sagittal spinal balance after surgery: Group A (Fig. 1B1, B2 and B3, one typical case in group A) with SVA ≥ 5 cm, or PT ≥ 20°, or PI-LL ≥ 20° and Group B (Fig. 1C1, C2 and C3, one typical case in group B) with normal sagittal alignment.

### Muscle parameters

All the muscular assessments were measured through the picture archiving and communicating system (PACS) in our hospital on lumbar MRI performed within 1 month before surgery. The following five muscle compartments were separately segmented from the upper endplate level of L1 to L5. Measurements of paraspinal muscle were obtained from T2-weighted images by the Image J software [17]. The parameters of muscle (Fig. 1C3) included total cross-sectional area (tCSA), functional cross-sectional area (fCSA, the area of lean muscle tissue), and fatty infiltration (FI, the ratio of fat area to tCSA). The fCSA of muscle was measured by the thresholding technique. rCSA was calculated by fCSA ratio to correspond level of lumbar CSA to eliminated personal difference [18].

### Statistical analysis

All statistical analyses were performed using the Statistical Package for the Social Sciences (SPSS, version 26.0, SPSS, Inc, Chicago, IL, USA). The normality of the data was tested by the Shapiro–Wilk test and variability by the Leneve test. Descriptive statistics were expressed as the means and standard deviation (SD). The characteristics of patients with sagittal imbalance were compared with those of patients without malalignment via the independent t-test or Wilcoxon rank-sum test for continuous variables. Pearson’s correlation analysis was used to analyze the correlation among the demographic, the sagittal spinal, and the muscle parameters and logistic regression analysis was used to identify the risk factors for postoperative sagittal malalignment. A *p*-value < 0.05 was statistically significant.

## Results

A total of 82 patients (male 25, female 57) were enrolled (Table 1). The proportion of males in group A was significantly lower than that in group B (*p* = 0.002). The age of Group A (73.36 ± 8.02) was significantly greater than that of Group B (69.08 ± 6.07, *p* = 0.009). There were no significant differences in BMI between Group A (25.48 ± 3.81) and B (26.39 ± 3.45, *p* = 0.263) and in the length of follow-up (8.84 ± 6.62 vs. 8.65 ± 5.26, *p* = 0.887).

**Table 1:** Comparison of preoperative demographic characteristics between group A and B

	Group A (*n* = 44)	Group B (*n* = 38)	*p* value
Sex (male)	7	18	0.002^a^
Age (year)	73.36 ± 8.02	69.08 ± 6.07	0.009^a^
BMI (kg/m^2^)	25.48 ± 3.81	26.39 ± 3.45	0.263
Follow up (month)	8.84 ± 6.62	8.65 ± 5.26	0.887

*BMI* Body mass index;

^a^Statistical significance at the level of 0.05

Preoperative SVA (4.79 ± 4.36 vs. 6.49 ± 3.68, *p* = 0.062), TK (33.50 ± 12.51 vs. 34.05 ± 12.01, *p* = 0.840), LL (38.68 ± 12.23 vs. 40.16 ± 13.89, *p* = 0.611) and SS (29.00 ± 7.43 vs. 32.27 ± 10.38, *p* = 0.102) didn’t displayed significant differences between groups (Table 2). Preoperative PI (56.53 ± 8.95), PT (27.40 ± 5.82), PI-LL (17.85 ± 10.57), and PTr (0.48 ± 0.09) in group A were significantly greater than those in group B (51.51 ± 9.70, *p* = 0.017; 19.30 ± 7.32, *p* < 0.001; 11.36 ± 11.44, *p* = 0.009; 0.38 ± 0.16, *p* = 0.001; respectively).

**Table 2:** Comparison of preoperative radiographic parameters between group A and B

	Group A (*n* = 44)	Group B (*n* = 38)	*p* value
SVA (cm)	4.79 ± 4.36	6.49 ± 3.68	0.062
TK (°)	33.50 ± 12.51	34.05 ± 12.01	0.840
LL (°)	38.68 ± 12.23	40.16 ± 13.89	0.611
SS (°)	29.00 ± 7.43	32.27 ± 10.38	0.102
PI (°)	56.53 ± 8.95	51.51 ± 9.70	0.017^a^
PT (°)	27.40 ± 5.82	19.30 ± 7.32	< 0.001^a^
PI-LL (°)	17.85 ± 10.57	11.36 ± 11.44	0.009^a^
PTr	0.48 ± 0.09	0.38 ± 0.16	0.001^a^

*SVA* Sagittal vertical axis, *TK* Thoracic kyphosis, *LL* Lumbar lordosis, *SS* Sacral slope, *PI* Pelvic incidence, *PT* Pelvic tilt, *PI-LL* Pelvic incidence minus lumbar lordosis, *PTr* Pelvic tilt and pelvic incidence ratio;

^a^Statistical significance at the level of 0.05

In group A, the tCSA (39.89 ± 5.45), the fCSA (28.73 ± 4.23), and the rCSA (2.78 ± 0.52) were significantly lower than those in group B (47.57 ± 8.41, *p* < 0.001; 36.94 ± 7.81, *p* < 0.001; 3.11 ± 0.63, *p* = 0.013; Table 3). The FI of group A (27.16% ± 5.58%) was significantly greater than that of group B (22.61% ± 5.81%, *p* = 0.001).

**Table 3:** Comparison of preoperative paraspinal muscle parameters between group A and B

	Group A (*n* = 44)	Group B (*n* = 38)	*p* value
tCSA (cm^2^)	39.89 ± 5.45	47.57 ± 8.41	< 0.001^a^
fCSA (cm^2^)	28.73 ± 4.23	36.94 ± 7.81	< 0.001^a^
rCSA	2.78 ± 0.52	3.11 ± 0.63	0.013^a^
FI (%)	27.16 ± 5.58	22.61 ± 5.81	0.001^a^

*tCSA* Total cross-sectional area, *fCSA* Functional cross-sectional area, *rCSA* Ratio of fCSA to the corresponding lumbar cross-sectional area, *FI* Fatty infiltration;

^a^Statistical significance at the level of 0.05

Postoperative SVA (3.30 ± 3.54), PI (56.06 ± 9.03), PT (26.72 ± 5.58), PI-LL (14.73 ± 8.57), PTr (0.48 ± 0.09) in group A were significantly greater than those in Group B (1.88 ± 2.53, *p* = 0.043; 51.34 ± 9.08, *p* = 0.021; 15.78 ± 3.81, *p* < 0.001; 2.79 ± 9.02, *p* < 0.001; 0.31 ± 0.08, *p* < 0.001; respectively; Table 4). Postoperative LL (41.33 ± 10.70) and SS (29.24 ± 7.54) in group A were significantly lower than those in group B (48.54 ± 11.07, *p* = 0.004; 35.39 ± 8.11, *p* = 0.001). Postoperative TK (34.81 ± 11.20 vs. 36.22 ± 12.85, *p* = 0.595) was not statistically different between groups.

**Table 4:** Comparison of postoperative radiographic parameters between group A and B

	Group A (*n* = 44)	Group B (*n* = 38)	*p* value
SVA (cm)	3.30 ± 3.54	1.88 ± 2.53	0.043^a^
TK (°)	34.81 ± 11.20	36.22 ± 12.85	0.595
LL (°)	41.33 ± 10.70	48.54 ± 11.07	0.004^a^
SS (°)	29.24 ± 7.54	35.39 ± 8.11	0.001^a^
PI (°)	56.06 ± 9.03	51.34 ± 9.08	0.021^a^
PT (°)	26.72 ± 5.58	15.78 ± 3.81	< 0.001^a^
PI-LL (°)	14.73 ± 8.57	2.79 ± 9.02	< 0.001^a^
PTr	0.48 ± 0.09	0.31 ± 0.08	< 0.001^a^

*SVA* Sagittal vertical axis, *TK* Thoracic kyphosis, *LL* Lumbar lordosis, *SS* Sacral slope, *PI* Pelvic incidence, *PT* Pelvic tilt, *PI-LL* Pelvic incidence minus lumbar lordosis, *PTr* Pelvic tilt and pelvic incidence ratio;

^a^Statistical significance at the level of 0.05

**Fig. 2:**
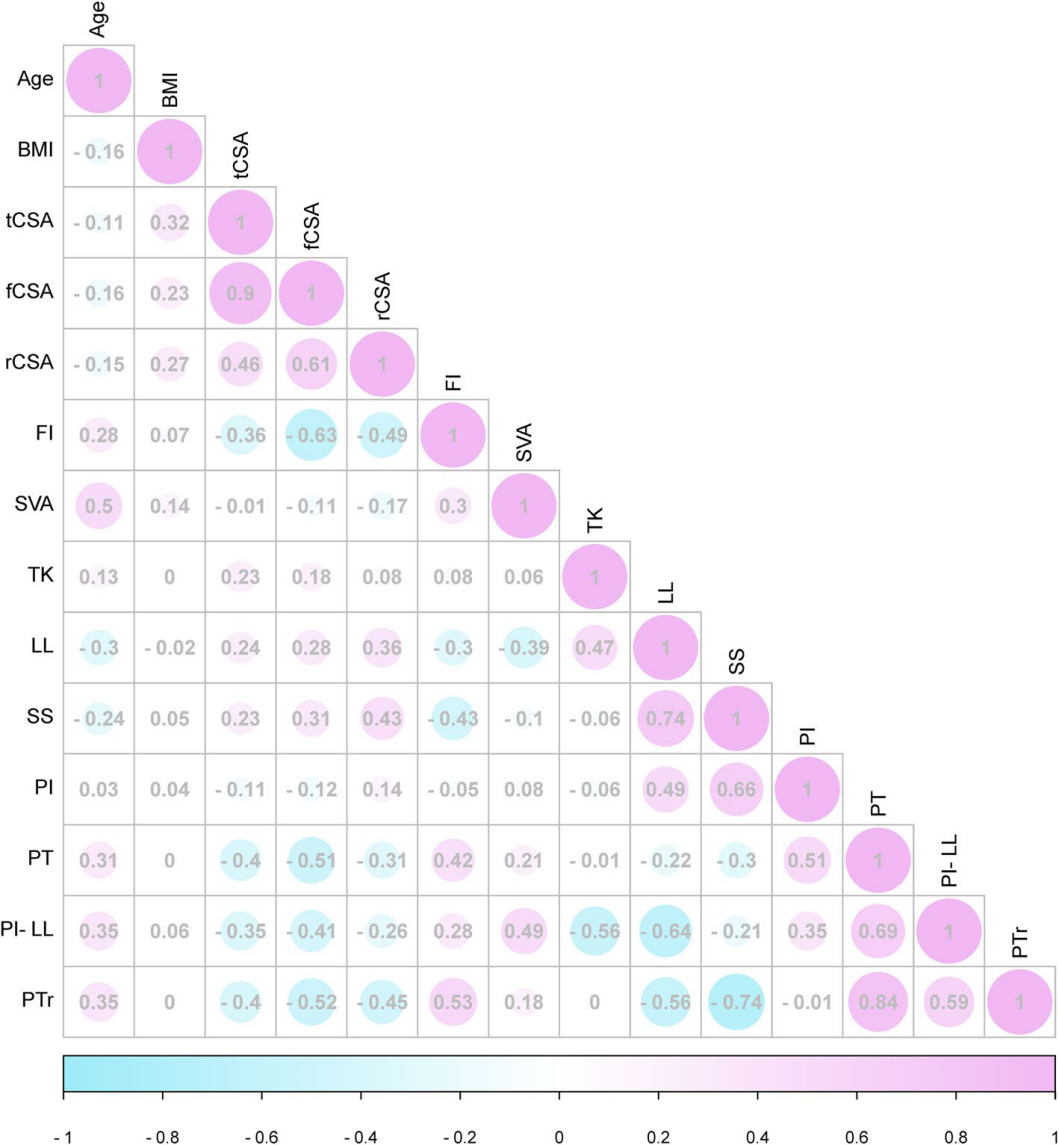
The correlation analysis among the demographic, the sagittal spinal, and the muscle parameters, with a greater radius representing a greater correlation coefficient

**Fig. 3:**
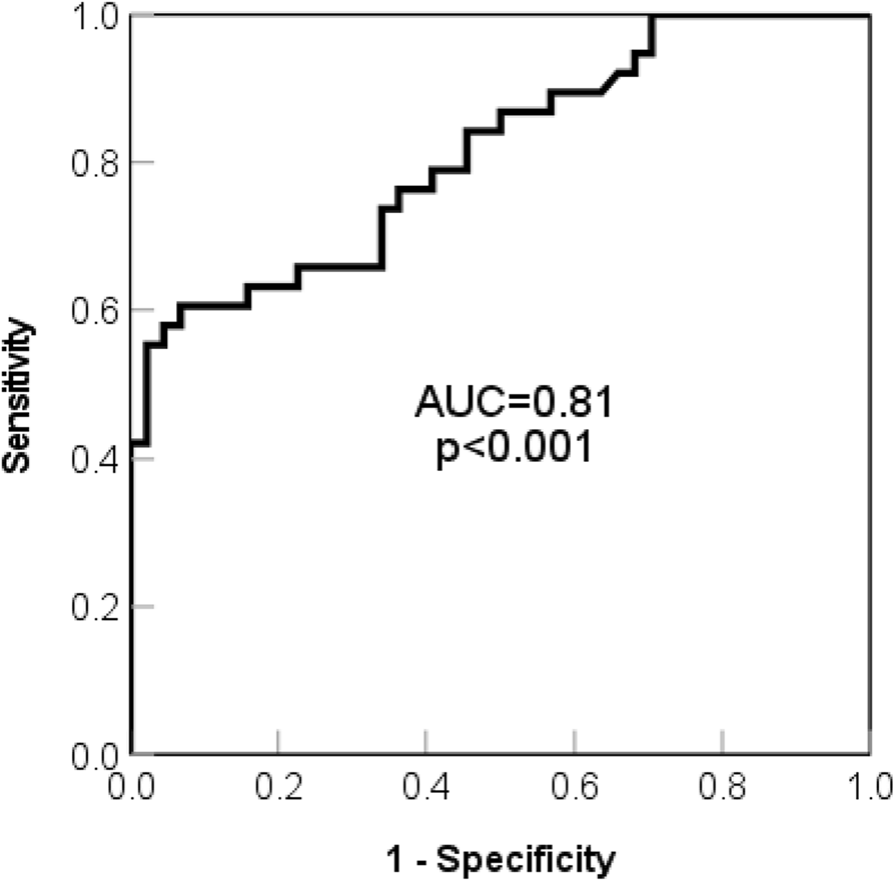
The receiver operating characteristic (ROC) analysis between fCSA and the postoperative imbalance, with the area under curve (AUC) of 0.81

Age was positively correlated with FI, SVA, PT, PI-LL and PTr, but negatively correlated with LL and SS (*p* < 0.05, Fig. 2). fCSA was negatively correlated with PI-LL, PT and PTr, and positively correlated with LL and SS (*p* < 0.05). FI was positively correlated with SVA, PT, PI-LL and PTr, and negatively correlated with LL and SS (*p* < 0.05). In logistic regression analysis (Table 5), statistically significant effect of fCSA (*p* = 0.003 OR = 0.797) and preoperative PT (*p* = 0.007 OR = 1.200) on postoperative sagittal malalignment. There was no statistically significant effect of sex (*p* = 0.684 OR = 0.674), age (*p* = 0.185 OR = 1.061), PI (*p* = 0.912 OR = 1.004) and PI-LL (*p* = 0.758 OR = 0.989) on postoperative sagittal malalignment. Figure 3 shows the ROC curve analysis between fCSA and postoperative imbalance, with the area under the curve (AUC) of up to 0.81 (*p* < 0.001).

**Table 5:** The logistic regression analysis of preoperative factors related to postoperative malalignment

	B	S.E	Wald	*p* value	OR	95% CI
Sex	-0.395	0.970	0.165	0.684	0.674	0.101 ~ 4.513
Age	0.059	0.045	1.758	0.185	1.061	0.972 ~ 1.158
fCSA	-0.227	0.076	8.973	0.003^a^	0.797	0.686 ~ 0.924
PT	0.183	0.068	7.301	0.007^a^	1.200	1.051 ~ 1.370
PI	0.004	0.038	0.012	0.912	1.004	0.932 ~ 1.082
PI-LL	-0.011	0.034	0.095	0.758	0.989	0.925 ~ 1.058
Constant	-0.881	4.823	0.033	0.855	0.414	

*fCSA* Functional cross-sectional area, *PT* Pelvic tilt, *PI* Pelvic incidence; *PI-LL* Pelvic incidence minus lumbar lordosis;

^a^Statistical significance at the level of 0.05

## Discussion

This study evaluated the predictors of the restoration of the sagittal spinal balance after short-segment decompression and fusion surgery in the elderly patients who had LLS with sagittal malalignment. 38 out of 82 patients recovered the sagittal morphology three months or later after surgery. Three types of preoperative predictors for the restoration of the sagittal spinal malalignment were found in this study, including the mass of lumbar paraspinal muscles, the sagittal radiographic spinal parameters, and some demographic characteristics.

LSS is caused by various factors, such as facet joint hypertrophy, osteophyte, or ligamentum flavum (LF) thickening [4, 19]. The LF is stretched during lumbar flexion, and more than half of LSS patients might assume a forward-bending posture to reduce pain during standing, which would lead to sagittal malalignment [20, 21]. After decompression and fusion of the stenosis levels, the trunk would no longer need to maintain an anterior trunk flexion position to relieve nerve compression. Therefore, observing symptom changes in a spine-extended position is a significant and effective way to determine whether the imbalance is caused by pain alleviation, which is also an important factor in the recovery of normal sagittal morphology postoperatively. Previous studies also demonstrated that the trunk muscle weakness led to adverse clinical outcomes in LSS patients [22, 23]. Our study confirmed that patients with a greater CSA of paraspinal muscle were more likely to restore the normal sagittal spinal alignment after short-segment fusion surgery. This might be related to the fact that the stronger paravertebral muscles would help to stabilize the trunk, maintain the proper posture, and generate movement, especially the multifidus and erector muscles [24]. Shen et al. [22] found that the sagittal malalignment was associated with the loss of the back muscle mass, especially the paravertebral muscles instead of the appendicular skeletal muscle weakness. Therefore, the paravertebral muscles were more important in the pathogenesis of sagittal malalignment in LSS patients.

In this study, the CSA of the lumbar paravertebral muscle at five levels from L1 to L5 were separately measured and then averaged as an indicator of muscle mass. Previous studies only measured the CSA of one single level [25], which lacked reliability in the assessment of the overall lumbar muscle. Functional CSA is a better indicator of the actual mass of muscles than the total CSA [18]. Therefore, this study could provide a reliable preoperative evaluation index for spinal surgeons. In addition, fCSA could better predict the occurrence of postoperative malalignment, and the AUC is up to 0.81. Nevertheless, the cutoff value could not be obtained due to the small sample size.

Legaye et al. [26] found that PI was almost a fixed value after adulthood despite a slight change as the sacroiliac ligament complex relaxed in the process of degeneration, and everyone had a matched LL. Through a large database study, Roussouly et al. [27] demonstrated that a greater PI was presented with a greater LL. Correspondingly, it required stronger muscle strength to maintain the LL. As the infiltration of fat in muscles and the reduction of fCSA, the muscle strength weakens, which would lead to reduction in LL, and anterior inclination of the trunk. The pelvis would tilt posteriorly for compensation, causing an increase in PT. Therefore, the fCSA of lumbar paraspinal muscle was negatively correlated with the postoperative PT.

PT is a more sensitive indicator in detecting the sagittal malalignment than SVA because the SVA could be maintained as a normal distance by pelvic retroversion. Previous studies have stated that PT is significantly correlated with VAS and ODI scores of patients [28]. When SVA is still in the normal range, sagittal malalignment has already occurred in the body, so PT is of great significance for the early patients in the evaluation of sagittal malalignment. Similarly, patients with a great PI might have a considerable PT. Therefore, to eliminate this effect, we calculated the ratio of PT to PI (PTr) and achieved similar result [16]. This is the first time to confirm a negative correlation between PT or PTr and fCSA after short-segment fusion surgery, which provides insight for application in the clinical setting.

Sex is another factor in influencing the trunk muscle strength as females experience a dramatic decline in hormones due to menopause at the beginning of sixties [29]. In this research, female patients were also tending to maintain the sagittal spinal malalignment after surgery, which attribute to the lower fCSA and the higher FI in females [30]. Therefore, when we make surgery strategy in elderly LSS patients with sagittal malalignment, the sex difference should be taken into consideration.

Our study also found that age was another risk factor for the failure to restore the normal sagittal spinal morphology after short-segment decompression and fusion surgery. This might be related to the muscle degeneration during the aging process. With an increase age, the paravertebral muscle would worsen, the LL would reduce, and the PT would increase to compensate for the increase of SVA. When these compensation mechanisms are exhausted, it would ultimately lead to the onset of sagittal malalignment. Previous study also found that a decrease in CSA of the paravertebral muscles and an increase in fatty infiltration with aging [31, 32], which is consistent with the results of the present study. Hence, in the elderly LSS patients with sagittal malalignment, the short-segment decompression and fusion surgery might not be the optimal option unless the muscle condition would be ameliorated.

Hence, we must declare that during the degenerative process, the paravertebral muscles, the spinal radiographic parameters, and the demographic characteristics of patients interact with each other. The debut factor and sequence of occurrence needs to be further investigated in future studies.

The limitations of present study are as follows: firstly, we included a relatively small sample size and did not derive cutoff values for fCSA of the lumbar paravertebral muscles in patients with postoperative imbalance. Prospective studies are needed to determine which factors would facilitate patients to regain the normal sagittal morphology after short-segment fusion. Secondly, our follow-up periods were relatively short, ranging from 3 to 30 months, but previous studies have shown that 3 months were sufficient to return most patients to normal sagittal alignment after surgery [33].

## Conclusion

Despite the limitations, we concluded that the fCSA was negatively associated with postoperative PT and PTr. A greater CSA of lumbar paraspinal muscles (especially fCSA), smaller preoperative PI, PT or PI-LL, males, and younger age are the predictors for the restoration of the sagittal spinal malalignment in the elderly patients with LSS after short-segment decompression and fusion surgery. These results could provide insights for developing the subject-specific treatment strategy for LSS patients with sagittal spinal malalignment.

## Data Availability

The datasets generated and/or analyzed during the current study are not publicly available due to data privacy rules but are available from the corresponding author on reasonable request.

## Abbreviations

LSS: Lumbar spinal stenosis
SVA: Sagittal vertical axis
TK: Thoracic kyphosis
LL: Lumbar lordosis
SS: Sacral slope
PI: Pelvic incidence
PT: Pelvic tilt
PI-LL: Pelvic incidence minus lumbar lordosis
PTr: Pelvic tilt and pelvic incidence ratio
tCSA: Total cross-sectional area
fCSA: Functional cross-sectional area
FI: Fatty infiltration
